# Ratio of Deaths, Insanity, Blindness, Etc., in Different Parts of the United States

**Published:** 1853-03

**Authors:** 


*Ratio of deaths, insanity, blindness, etc., in different parts of the United States.* — For the following statistics from the last national census, we are indebted to the New York Medical Times:

| | Annual deaths per cent. | Ratio to the number living. |
|---|---|---|
| New England States, . . . . | 1.55 | 1 to 64 |
| Middle States, with Ohio, . . . . | 1.39 | 1 to 72 |
| Central Slave States, . . . . | 1.38 | 1 to 73 |
| Coast Planting States, . . . . | 1.37 | 1 to 73 |
| North Western States, . . . . | 1.24 | 1 to 80 |
| United States, total, . . . . . | 1.38 | 1 to 73 |

From the same source are derived the following statistics:

There are 9,091 white *mutes* in the United States, and 632 colored, of whom 489 are slaves. Among the white population, there is one deaf mute to each 2,151; of the free colored, one to each 3,005, and among the slaves, one to each 6,552. There are 9,702 *blind* persons, of whom 7,997 are white, 1,211 slaves, and 494 free colored persons. Among the whites, there is one blind person to each 2,445; among the free colored, one to each 870; among the slaves, one to each 2,645. Of *insane* persons the whole number is 15,768, of whom 15,156 are whites, 321 free colored, and 291 slaves. Of *idiots*, there are whites, 14,230; free colored, 436; slaves, 1,040. With the white population in the United States, there exists one insane person for each 1,280 individuals; among the free colored, one to each 1,338; and among the slaves, one to each 11,010. With respect to idiocy, the white population presents one to each 1,374 persons; the free colored, one to each 985; and among the slaves one to each 3,080.

